# Immunohistochemical expression of glucose transporter 1 in keratin-producing odontogenic cysts

**DOI:** 10.1186/s12903-016-0191-2

**Published:** 2016-03-10

**Authors:** Beatriz Vera-Sirera, Leopoldo Forner-Navarro, Francisco Vera-Sempere

**Affiliations:** Department of Stomatology, University of Valencia, Valencia, Spain; Department of Stomatology, Endodontics Unit, University of Valencia, Valencia, Spain; Department of Pathology, University of Valencia and La Fe University Hospital, Valencia, Spain

**Keywords:** Glucose transporter protein, Immunohistochemistry, Keratin-producing odontogenic cyst, Keratocystic odontogenic tumour, Orthokeratinised odontogenic cyst, Positron emission tomography

## Abstract

**Background:**

Keratin-producing odontogenic cysts (KPOCs) are a group of cystic lesions that are often aggressive, with high rates of recurrence and multifocality. KPOCs included orthokeratinised odontogenic cyst (OOC) and parakeratotic odontogenic cysts, which are now considered true tumours denominated keratocystic odontogenic tumours (KCOTs). GLUT1 is a protein transporter that is involved in the active uptake of glucose across cell membranes and that is overexpressed in tumours in close correlation with the proliferation rate and positron emission tomography (PET) imaging results.

**Methods:**

A series of 58 keratin-producing odontogenic cysts was evaluated histologically and immunohistochemically in terms of GLUT1 expression. Different data were correlated using the beta regression model in relation to histological type and immunohistochemical expression of GLUT1, which was quantified using two different morphological methods.

**Results:**

KPOC cases comprised 12 OOCs and 46 KCOTs, the latter corresponding to 6 syndromic and 40 sporadic KCOTs. GLUT1 expression was very low in OOC cases compared with KCOT cases, with statistical significant differences when quantification was considered. Different GLUT1 localisation patterns were revealed by immunostaining, with the parabasal cells showing higher reactivity in KCOTs. However, among KCOTs cases, GLUT1 expression was unable to establish differences between syndromic and sporadic cases.

**Conclusions:**

GLUT1 expression differentiated between OOC and KCOT cases, with significantly higher expression in KCOTs, but did not differentiate between syndromic and sporadic KCOT cases. However, given the structural characteristics of KCOTs, we hypothesised that PET imaging methodology is probably not a useful diagnostic tool for KCOTs. Further studies of GLUT1 expression and PET examination in KCOT series are needed to confirm this last hypothesis.

## Background

Keratin-producing odontogenic cysts (KPOCs) form a heterogeneous group of cystic lesions that are often aggressive in character, with high rates of recurrence and multifocality [1], associated to a marked proliferative activity [2, 3]. The lesional spectrum of KPOCs includes primarily orthokeratinised odontogenic cysts (OOC) and odontogenic keratocysts, which, according to WHO guidelines [4] are also referred to as keratocystic odontogenic tumours (KCOTs), in accordance with KCOTs being true tumoural growths. Effective management of these cystic lesions is subject to frequent discussion [5] and malignant transformation is possible, albeit very rare [6].

Glucose, as a primary energy source, enters intracellularly by a family of transporters called GLUTs, of which at least 13 members are now know with cell-specific expression [7]. Within these families, GLUT1 or erythrocyte glucose transporter is over-expressed in tumours, particularly in malignant lesions, as glucose is the main energetic source of these neoplastic growths [8]. This is based on use of an imaging methodology, so-called positron emission tomography (PET) alone or combined with computed tomography (PET-CT), that used as radiotracer the glucose analogue 18F-fluorodeoxyglucose (18FGD) [9].

In head-and-neck area, GLUT1 was also overexpressed at particular times in some benign tumours [10, 11], particularly those with marked proliferative activity [12]. Likewise, in some benign odontogenic tumors with high recurrence rates, it has been noted that GLUT1 overexpression, to the extent that it may be useful for PET or PET-CT to assess the recurrence of these benign tumours [13]. However, in our knowledge, GLUT1 expression has not been investigated in KPOCs with a high rate of recurrence, which are presently regarded as a true odontogenic tumours with a high probability of relapse [1, 4, 5].

In the present study, we aimed to analyse, for the first time to our knowledge, GLUT1 expression in a series of KPCOs, to differentiate histological subtypes and evaluate the potential for use of PET in the diagnosis of these lesions, similar to what has been reported in other benign tumours of the head and neck with high recurrence rates [13].

## Methods

We studied 58 cases of KPOCs that were diagnosed over a period of 10 years (2005-2015) at the Department of Pathology of La Fe University Hospital, Valencia, Spain. Histological material was retrieved from storage. Our work formed part of a project previously approved by our Institutional Review Board (Comité Ético de Investigación Clínica -CEIC-) (protocol no. 2013/0045) and the guidelines outlined in the Declaration of Helsinki, as revised in Séul -Corea- 2008 were strictly followed. Prior to performing oral surgery, written informed consent was obtained in all patients, communicating the participation of its oral biopsy specimen in this immunohistochemical study. We selected cases using a pathological diagnosis data base (Pat Win® version 4.1.4). We performed a 10-year retrospective search employing the search terms “keratocyst”, “primordial cyst”, “keratocystic odontogenic tumour”, “orthokeratinised odontogenic cyst”, and “keratinized cyst”, understanding the varied nomenclature [14] used in these lesions.

All original histological sections were reviewed microscopically by two observers (BVS, FVS), and were reclassified using the WHO 2005 guidelines (4) and diagnostic criteria outlined in the OOC [15, 16]. The clinical, radiological, and surgical data on all patients were gathered from the medical records using the Mizar® 2.0 platform with the aid of the viewfinder Luna® 3.0. We noted the following data: age; gender; location of the lesion (mandibular and/or maxillary); clinical/radiological follow-up findings, number of recurrences; and any clinical, pathological, or genetic data suggesting a syndromic association. We used the criteria of Kimonis et al. [17] to diagnose nevoid basocellular carcinoma syndrome (NBCCS).

Sections 5 μm in thickness were cut from the original paraffin-embedded blocks and mounted on poly-Llysine- coated glass slides prior to immunohistochemical staining. Epitope retrieval proceeded at 97°C for 20 min in high-pH EnVision FLEX Target Retrieval solution, followed by washing for 5 min in EnVision FLEX Wash Buffer. GLUT 1 was immunostained using a concentrated polyclonal antibody (Master Diagnostica, Granada, Spain), at 1/50 dilution. The incubation time was 20 min and staining was visualized using the high-pH EnVision FLEX system. Placental sections and erytrocytes membranes served, respectively, as external and internal, positive staining controls and the negative controls were mock-stained test sections (the primary antibody was replaced by phosphate-buffered saline –PBS-).

GLUT 1 immunostaining at epithelial level of KPOC was quantified using the Leica DMD108 digital microscopy system (Leica Microsystems^©^, Wetzlar, Germany) and two different procedures. First, the thickness (values from 0 to 100 %) of the epithelium marked by GLUT1 was measured. Second, an immunoreactivity score for GLUT1 (IRS-GLUT1), based on the IRS described by Remmele et al. [18], was established. IRS assesses two parameters: immunostaining intensity (0 = absent; 1 = weak; 2 = moderate; 3 = strong) and percentage of labelled cells (values from 0 to 4: 0 = 0 %; 1 = 1-25 %; 2 = 25-50 %; 3 = 50-75 %; 4 = 75-100 %). Product of both parameters supplies the IRS (values from 0 to 12). Additionally, location of immunostaining (basal, suprabasal, intermediate or superficial) was recorded.

The results obtained were expressed as the mean, standard deviation (sd), median and interquartile range for each lesional type. To contrast the differences in the expression of GLUT1, the percentage thickness of the epithelium stained and IRS score were compared, using the beta regression model, rescaling the variables "score" and “percentage thickness stained” at range [0-1]. Statistical analysis was performed using the statistical software R, version 3.1.2, considering a P value less than 0.05 as indicative of statistical significance.

## Results

Upon histological review, the 58 KPOCs were classified, using the WHO criteria, into 46 KCOTs and 12 OOCs. Of the 46 KCOT cases, 6 were considered to be syndromic KCOT (S-KCOT) because other lesions or abnormalities consistent with the presence of NBCCS were also noted. Table 1 summarises the epidemiological data, as well as the frequency of recurrences, based on information from the clinical and radiological follow-up. Notably, in no case in our series was imaging diagnosis performed using PET.

**Table 1:** Epidemiological and follow-up data

*Lesional type*	*N° cases*	*Mean age (years)*	*M/F ratio*	*Follow-up* ^a^	*% Recurrence*	*Location* ^b^		
						Mb	-	83,33 %
OOC	12	35,50 ± 12,10	7/5	39,0 ± 26,01	0 %	Mx	-	16,66 %
						Mb	-	75,00 %
NS-KCOT	40	45,67 ± 21,20	21/19	29,6 ± 31,04	35 %	Mx	-	22,50 %
						Mb & Mx	-	2,50 %
						Mb	-	33,33 %
S-KCOT	6	23,00 ± 18,07	1/5	112,0 ± 76,10	100 %	Mx	-	16,33 %
						Mb & Mx	-	50,00 %

^a^ *Clinico-radiological mean follow-up expressed in months* ^b^ *Mb: mandibular; Mx: maxillar*

S-KCOT (six cases) affected five females and one male, aged 9–54 years, and the mean patient age at the time of diagnosis was 23 ± 18.07 years. All patients had recurrences; the mean number of recurrences was 3 ± 1.41 over a mean follow-up time of 112 ± 76.1 months. Lesions occurred in the mandible and maxilla (three patients), mandible alone (two patients), and maxilla alone (one patient). The premolar region was the most frequently affected, followed by the molar and anterior regions.

Non-syndromic KCOT (NS-KCOT; 40 cases) occurred in 21 males and 19 females (M/F ratio: 1.10) with a mean age of 45.67 ± 21.2 years (range, 9–84 years), and 35 % of patients (14 of 40) had recurrences (an average of 1.4 ± 0.6 relapses) over 29.6 ± 31.04 months of follow-up. Of all patients, 75 %, 22.5 %, and 2.5 % had lesions in the mandible, maxilla, or both, respectively. The premolar mandibular region was most frequently affected, and the anterior region least affected.

OOCs were noted in seven males and five females, and the mean age at diagnosis was 35.5 ± 12.1 years (range, 19–65 years). Of all patients, 83.5 % had mandibular involvement exclusively, and only two had lesions in the maxilla. The molar mandibular area, followed by the premolar mandibular region, was the most frequently affected site. Five of the twelve cysts (41.6 %) were associated with impacted teeth and were initially clinically and radiologically misdiagnosed as dentigerous cysts. No OOC patient had multiple lesions, and no patient had recurrence or developed of another lesion during a mean follow-up time of 39 ± 26.1 months (range, 9–95 months), confirming a significant difference (*p* = 0.039) compared with OOC against all KPOCs in relation to recurrence.

**Fig. 1:**
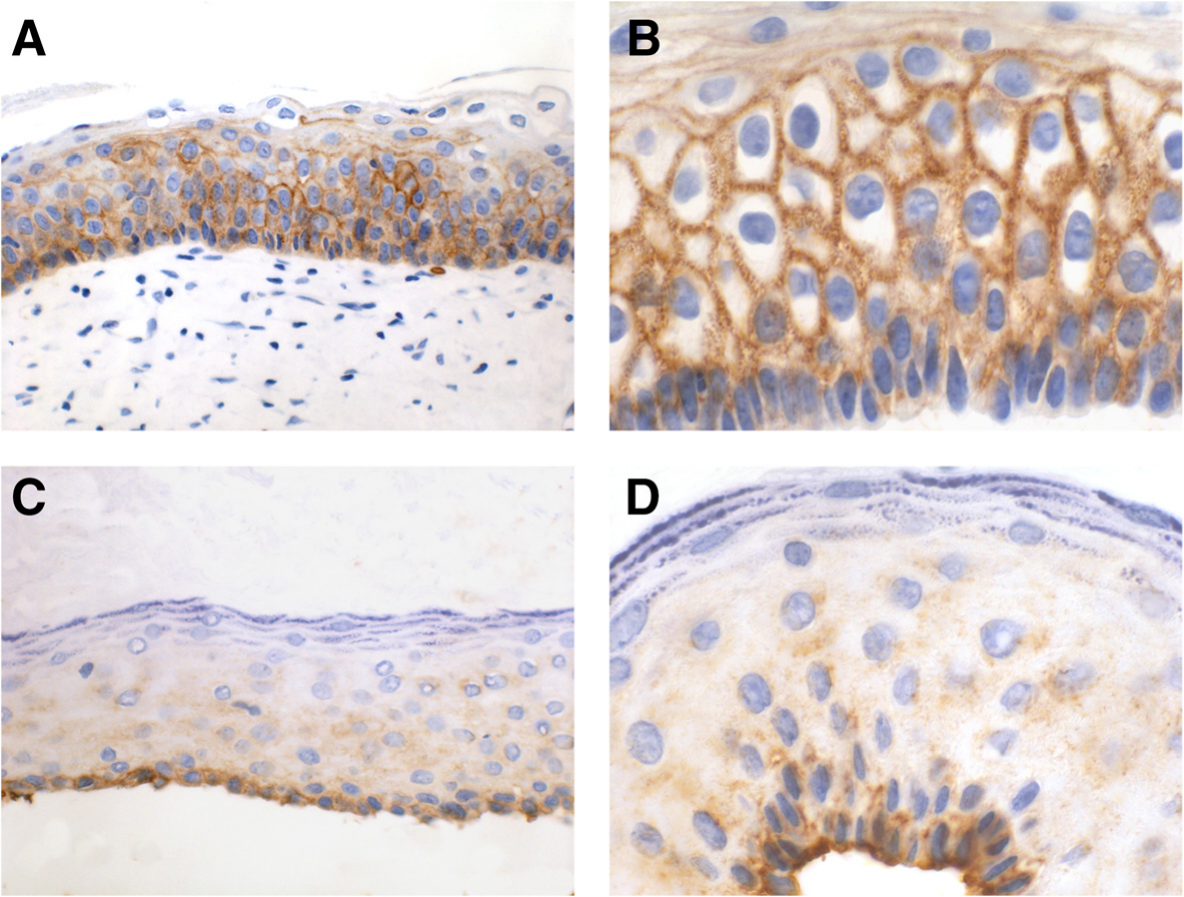
GLUT1 expression in KCOT and OOC. KCOT (a and b) showing a pronounced GLUT1 immunostaining with a predominantly membranous pattern (**a**). Immunoreactivity is especially marked in the parabasal and intermediate epithelium levels (**b**). Weak GLUT1 immunostaining in OOC (**c** and **d**), with a granular cytoplasmic and also slightly membranous pattern, somewhat more pronounced at basal level (**d**). (GLUT1, 200x and 400x)

**Fig. 2:**
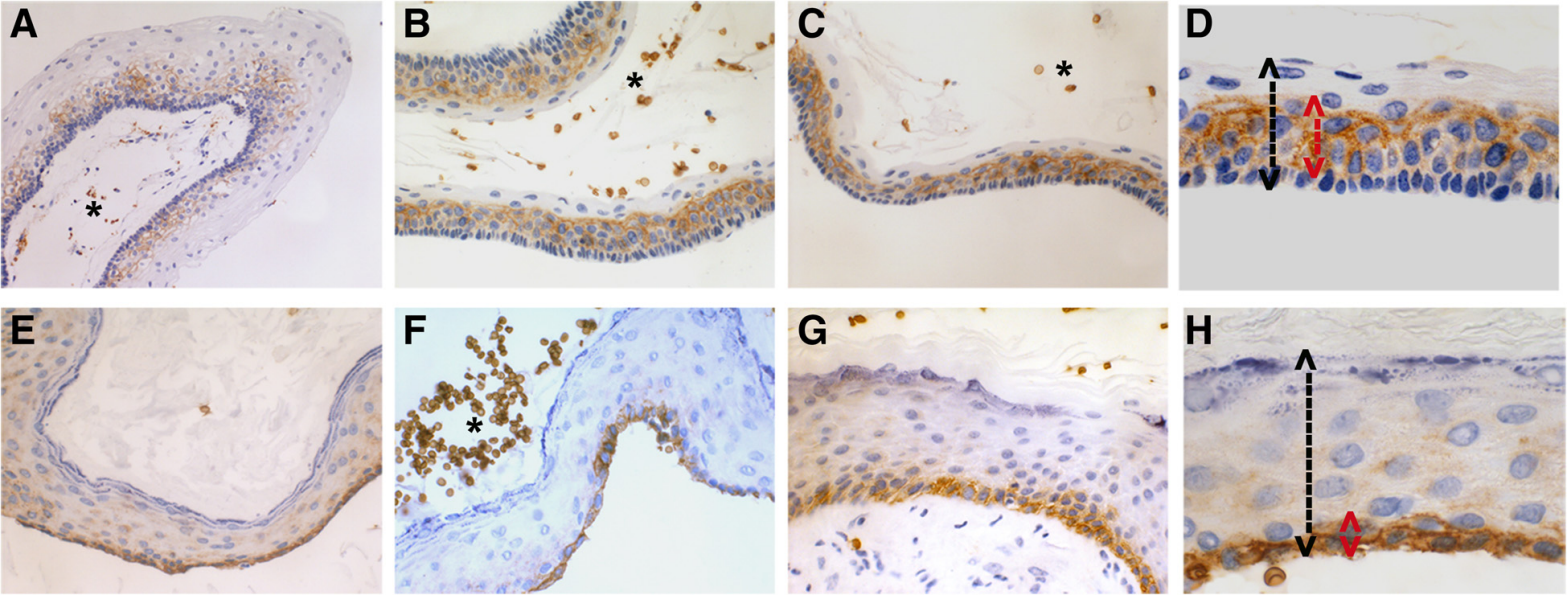
GLUT1 expression in KCOT and OOC. Panoramic view and details of GLUT1 immunostaining in KCOT (**a,b,c** and **d**) and OOC samples (**e,f,g** and **h**), displaying the different proportion of the epithelial thickness with GLUT1 immunostaining. Erythrocytes (asterisks) appears with an intense labelling, as an internal positive control. (GLUT1, 100, 200x and 400x)

After performing immunohistochemistry in our KPOC series (Figs. 1 and 2), we observed that GLUT1 expression was low and inconspicuous in OOC, showing only an weak cytoplasmic and membranous reactivity, that was distributed, in the basal, parabasal and intermediate layers of cystic epithelium, sometimes with a predominance in the basal reactivity. In contrast, immunoreactivity was greater in KCOT, with more pronounced membranous immunostaining in parabasal than in basal cells. Intermediate cells of KCOT were also labelled but with moderate immunostaining, often somewhat higher than basal cell. Furthermore, we observed that GLUT1 immunostaining was very similar, both in intensity and location, in S-KCOT and NS-KCOT cases.

Regarding quantification of GLUT1 expression, the IRS-GLUT1 (score with values ranging from 0 to 12) numerical results were generally low (Table 2), especially in OOC cases (mean score: 3.84 ± 2.93) compared with KCOT cases (mean score: 5.53 ± 2.62 ). Compared both mean scores, using the beta regression model, a statistical trend close to significance (*p* = 0.057) was found, in relation to the IRS of OOC group, which were lower those in the KCOT group. Likewise among the KCOT cases, the S-KCOT cases showed a slightly higher IRS (6.28 ± 2.13) that the NS-KCOT cases (IRS: 5.37 ± 2.72) and than the IRS values of both subgroups S-KCOT and NS-KCOT, albeit with no significant difference ( *p* = 0.477).

**Table 2:**
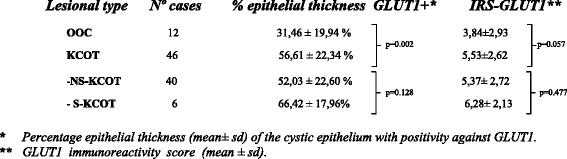
GLUT-1 expression in KPOC cases

By contrast, in assessing the expression by the epithelial thickness percentage GLUT1+ (Table 2) (Fig. 2), we found that OOC cases have a smaller thickness (31.46 ± 19.94 %) with GLUT1+ immunostaining than KCOT cases (56.61 ± 22.34 %), and the difference was statistically significant (*p* = 0.002). Thus, the analysis of the percentage thickness of the epithelium GLUT1+ staining allowed discrimination of the two KPOC subtypes. Finally, in S-KCOT subgroup, the percentage thickness with GLUT1+ immunostaining (66.42 ± 17.96 %) was higher than that in the sporadic (NS-KCOT) cases (52.03 ± 22.60 %), albeit with no significant difference (*p* = 0.128).

## Discussion

In the present study, we have analysed for the first time to our knowledge, the expression of GLUT1, a protein transporter involved in the active uptake of glucose across cell membranes, in a series of KPOCs, which constitute a heterogeneous group of cystic odontogenic lesions, that are often aggressive in character, with high rates of recurrence and multifocality [1], and are associated with a marked proliferative activity [2, 3].

GLUT1 expression has an important relationship with 18FGD-PET methodology, a rapidly developing functional-imaging modality that has shown great promise in the fields of primary, recurrent and metastatic tumour detection, as well as in the planning and monitoring of the therapy of various tumours [19].

GLUT1 or erythrocyte glucose transporter is the best-known member of the GLUTs family of insulin-independent transporters that are overexpressed in tumoural lesions [7], especially malignant ones, given the increased glucose consumption present in tumour cells needed to provide energy to cell proliferation [8]. GLUT1 expression in different models of tumour growth has been closely correlated, first, with the rate of cell proliferation, and second, with the effectiveness of 18FGD-PET methodology as diagnostic imaging technique [20], given that, like glucose, 18FDG passes through the cell membrane via GLUTs, and then is subjected to phosphorylation by hexokinase to form FDG-6-phosphate. FDG-6-phosphate is not metabolised further, and accumulates in tissues with active glucose metabolism [21].

Within the lesional spectrum of KPOC, cystic forms of relatively innocuous character have been identified, such as the so-called OOC [15, 16]; and also included aggressive forms, classically denominated odontogenic keratocysts [1] and today called KCOT [4]. These latter lesions show marked proliferative activity [2, 3] and have a high potential for recurrence [5], to the point of being considered true odontogenic tumour growth [4].

In our results we verified that the OOC present in the basal, parabasal and intermediate layers a very limited expression of GLUT1. Comparatively KCOT cases show a high reactivity, focused mainly on the layer of parabasal cells. Additionally, the expression levels of GLUT1 quantified in OOC and KCOT showed a statistically significant difference (or were close to statistical significance) according to the thickness of the cystic epithelium marked by GLUT1 or IRS-GLUT1, respectively. However, in our study, GLUT1 expression not discriminate between syndromic and sporadic cases of KCOT.

In tumours, GLUT1 expression is well correlated with cellular proliferative rates given the active glucose metabolism of highly proliferating tumours [21]. This relationship was confirmed in our study by the clear differences in the expression of GLUT in OOC and KCOT. Our results appears to be in agreement with those of other studies that reported obvious differences in the proliferation rates of both cystic odontogenic lesions, using various proliferative immunohistochemical markers, such as Ki 67 [2] or cyclin D1 [2, 3]. GLUT1 expression was established in our KCOT cases, with higher intensity, particularly at the parabasal cells level, and these data reinforced the role of the parabasal cells as protagonists elements in the tumour growth of KCOT [2, 3]. Considering that the type of treatment performed is known to have a remarkable influence in recurrence rates of KCOT [5], it should be noted that, given the retrospective character of our study, with various and non-homogeneous treatments, it is not possible to establish with certainty the relationship between GLUT1 expression and lesional recurrence.

Another aspect to consider is the relationship between the expression of GLUT1 and the efficacy of tumour detection by PET or combined PET-CT. Several immunohistochemical studies have demonstrated overexpression of GLUT1 in human malignant tumours and a correlation between GLUT1 expression and neoplastic progression [21]. Additionally, the overexpression of GLUT1 has been reported to be closely related to 18FGD uptake on PET, because 18FGD is also taken up via intracellular transport by GLUT1 [21–25].

Possible uses and applications of PET are paradoxically not limited to malignant tumours; thus, in the head-and-neck area, it has been noted that some benign tumours [26], such as pleomorphic adenoma [10], myofibroma [12] and ceruminous adenoma [11], demonstrate positivity by PET. These benign lesions are often included in the list of possible errors and pitfalls in the evaluation of head-and-neck cancers using PET but this correct knowledge can provide useful diagnostic information in an adequate clinical context [26].

Within odontogenic tumours, a previous study [13] showed four cases of ameloblastoma, a benign tumor with a high recurrence rate, with reactivity against GLUT1 and positivity in PET examination, postulating that PET-TAC can be useful in the imaging diagnosis of recurrences in ameloblastoma. In this report [13], GLUT1 immunostaining was not quantified, so it is not possible to compare our results in KCOT with those reported in ameloblastomas. On other hand, there is no published study regarding the use of PET-CT in the diagnosis or follow-up of KCOT, and in our series of KPOC, no case underwent PET or PET-CT examination.

However, despite this latter limitation, several points suggest that PET methodology is not a useful diagnostic tool in the case of KCOT. In first place, the mean IRS-GLUT1 found in our study was low (5.53 ± 2.62) on a possible maximum punctuation of 12, and a second aspect very important to consider is the tumour structure. Thus, KCOT is a cystic tumoural lesion where GLUT1 reactivity is limited to the epithelial lining. Otsuru et al. [13] reported a case of unicystic ameloblastoma and three non-unicystic types. Curiously, unicystic ameloblastoma showed lower reactivity to GLUT1 and lower 18FDG uptake compared with the solid tumour types [13]. This finding suggests that the effectiveness of 18FGD-PET is linked, regardless of the reactivity of GLUT1, to the volume of the tumoural mass [27]. Additionally, the hypothesis that PET-CT methodology is probably not useful in the case of KCOT, is established because the cellular uptake of 18FGD by KCOT did not reach levels sufficient to cause an adequate SUVmax (maximum standardised value). Further studies, analysing GLUT1 expression and PET in KCOT series, are necessary, in our opinion, to determine whether analysis of GLUT1 expression in ameloblastoma is applicable to KCOT and whether 18FGD-PET is a useful tool in the diagnosis of KCOT recurrence.

## Conclusions

GLUT1 expression differentiated between OOC and KCOT cases, with significantly higher expression in KCOTs, but did not differentiate between syndromic and sporadic KCOT cases. However, given the structural characteristics of KCOTs, we hypothesised that PET imaging methodology is probably not a useful diagnostic tool for KCOTs. Further studies of GLUT1 expression and PET examination in KCOT series are needed to confirm this last hypothesis.

## Abbreviations

GLUT1: erythrocyte glucose transporter 1
KPOC: keratin-producing odontogenic cysts
KCOT: keratocystic odontogenic tumour
OOC: orthokeratinised odontogenic cyst
NBCCS: nevoid basocellular carcinoma syndrome
PBS: phosphate-buffered saline
IRS: immunoreactive score
PET: positron emission tomography
CT: computed tomography
18FGD: 18F-fluorodeoxyglucose
WHO: World Health Organization
S-KCOT: syndromic keratocystic odontogenic tumour
NS-KCOT: non syndromic keratocystic odontogenic tumour
